# Effects of Sodium–Glucose Cotransporter 2 Inhibitors on Calcium Homeostasis: Where We Stand Now

**DOI:** 10.3390/cells14100724

**Published:** 2025-05-15

**Authors:** Alessandro Cuttone, Anastasia Xourafa, Carmela Morace, Vittorio Cannavò, Francesca Maria Bueti, Giuseppe Mandraffino, Giovanni Squadrito, Giorgio Basile, Agostino Gaudio, Antonino Catalano, Giuseppina Tiziana Russo, Federica Bellone

**Affiliations:** 1 Unit of Internal Medicine, Department of Clinical and Experimental Medicine, University Hospital G. Martino, University of Messina, 98100 Messina, Italy; alessandrocuttone@alice.it (A.C.); carmela.morace@unime.it (C.M.); vittorio.cannavo@hotmail.it (V.C.); francescabueti085@gmail.com (F.M.B.); giuseppe.mandraffino@unime.it (G.M.); giovanni.squadrito@unime.it (G.S.); giuseppina.russo@unime.it (G.T.R.); 2 Unit of Thalassemia, Policlinico “G. Rodolico”, Via S. Sofia 78, 95123 Catania, Italy; axourafa@gmail.com; 3 Unit of Geriatrics, Department of Biomedical and Dental Science and Morphofunctional Imaging, University Hospital G. Martino, University of Messina, 98100 Messina, Italy; giorgio.basile@unime.it; 4 Unit of Internal Medicine, Department of Clinical and Experimental Medicine, University of Catania, Policlinico “G. Rodolico”, 95123 Catania, Italy; agostino.gaudio@unict.it; 5 Unit of Geriatrics, Department of Clinical and Experimental Medicine, University of Messina, 98100 Messina, Italy; catalano.antonino@unime.it

**Keywords:** diabetes mellitus, SGLT2 inhibitors, empagliflozin, canagliflozin, dapagliflozin, ertugliflozin, calcium metabolism, FGF-23, parathyroid hormone, bone fragility

## Abstract

Diabetes mellitus has been knowingly associated with increased risk of fractures, so much so that skeletal fragility is considered a complication of diabetes. Determinants of bone fragility in this chronic condition are several, and the diabetes treatment choice could influence bone metabolism. Sodium-glucose cotransporter-2 inhibitors (SGLT2i) have recently expanded the therapeutic armamentarium for type 2 diabetes mellitus (T2D); these antihyperglycemic drugs act by reducing renal glucose reabsorption in the proximal tubule and have a proven cardiorenal benefit. The role of SGLT2i towards phospho-calcium metabolism is still unclear, so we aimed to review the current evidence of the relationship between SGLT2i and calcium and phosphate homeostasis. The PubMed, Scopus, and Web of Knowledge databases were searched to identify original research articles, meta-analyses, and scientific reviews on effects on bone metabolism in T2D patients treated with SGLT2i. Emerging data indicate that SGLT2i may lead to a rise of bone turnover markers, promoting a lower skeletal bone density and an increased fracture risk on murine models, but in real-world studies, results are controversial. Therefore, more clinical trials are needed to further clarify this topic, and the effects of SGLT2i on calcium homeostasis remain to date poorly understood.

## 1. Introduction

### 1.1. Diabetes and Bone Fragility

Diabetes mellitus is a chronic disease that affects around 537 million people aged 20–79 years around the world [1]. Uncontrolled hyperglycemia can lead to deleterious effects on several target organs such as the kidney, heart, brain, retina, and liver [2,3]. Among these, one of the complications on which the scientific literature is now putting the spotlight is the effect that this chronic disease has on bone metabolism [4].

Indeed, current literature data show that there is a strong interaction between insulin and bone metabolism [5]. It is therefore not surprising how its deficiency, such as in patients with type 1 diabetes (T1D), can lead to pathological effects on bone; particularly, when compared to subjects of the same age, Body Mass Index (BMI), and sex but without diabetes, patients with T1D have a lower level of bone mineral density (BMD) [6]. Underlying this low BMD appears to be insulin deficiency: this hormone, in fact, appears to stimulate mitosis in osteoblasts and inhibit their apoptosis [7,8]. In detail, insulin seems to act on three levels: by inhibiting p27, an inhibitor of cell proliferation in osteoblasts [9]; by phosphorylating Bcl-2 agonist of cell death (BAD) to block its apoptotic effects [10]; and, above all, by stimulating insulin-like growth factor 1 receptor (IGFR-1) [11]. Moreover, insulin deficiency, other factors seem to influence bone fragility in T1D patients. These include the presence of bone marrow fat, increased myokines, impairment of the RANKL/RANK axis, and the GH/IGF1 axis [12].

Whether this to date represents a milestone in T1D, in patients with type 2 diabetes (T2D) a relatively new condition known as ‘the diabetic bone paradox’ has been introduced: it consists of an increased risk of fracture in diabetic patients although BMD is often normal or, paradoxically, increased [13]. Underlying this paradox appears to be the increased bone porosity triggered by the consequences of hyperinsulinemia and especially hyperglycemia and amplified by the comorbidities commonly associated with T2D [14]. The presence of diabetic disease has shown to induce an increase in Bone Marrow Adipose Tissue (BMAT) not correlated with lower BMD; however, it was associated with an increased risk of fracture in mice models [15,16]. In humans, a study showed that obese patients had accelerated senescence of bone mineral microenvironment and that this contributed to an imbalance in the mineral properties of the bone in terms of increasing its cortical porosity [17,18].

Thus, meta-analyses in the literature show that the risk of hip fractures in men with T2D is 2.8 times higher than in the non-diabetic male population and 2.1 times higher in diabetic women than in non-diabetic women [19]. Another factor that seems to contribute to bone fragility appears to be the hyperglycemic state that typically characterizes T2D patients. This is linked to the presence of advanced glycation end products (AGEs) that also aggregate on type 1 collagen, interrupting the adhesion of osteoblasts to the extracellular matrix, increasing bone fragility [20]. Alteration of the extracellular matrix also leads to decreased alkaline phosphatase activity in osteoblasts, and this, of course, leads to alterations in bone mineralization [21]. Finally, the presence of the pro-inflammatory state produced by diabetes promotes bone resorption [22] and blocks the differentiation of osteoblasts [23]. This prolonged chronic inflammatory state and the spread of pro-inflammatory cytokines, which is a persistent characteristic of patients suffering from a chronic disease such as diabetes, could also have a role in the pathogenesis of diabetic bone disease [11]

Obesity represents another important feature that often accompanies T2D: the presence of visceral fat characterizes this pathological condition. Although they are produced by adipose tissue, adipokine levels in T2D patients are reduced [24]. Adipokines appear to have anabolic effects on osteoblasts, at least in vitro [25]. This, together with the low lectin levels that characterize diabetic patients, appears to adversely affect bone health [26].

Furthermore, according to the recent evidence available in the literature, it is noticeable that patients with T2D are characterized by a greater porosity of the cortical bone, seen both in studies involving the use of Magnetic Resonance Imaging (MRI) and of Quantitative Computed Tomography (QCT) [27,28].

In conclusion, some studies suggest that there is an impaired blood supply to the bone, particularly involving the cortical bone, which shows how the microvascular damage of diabetes may play a critical role in the deterioration of bone formation [11].

**Figure 1:**
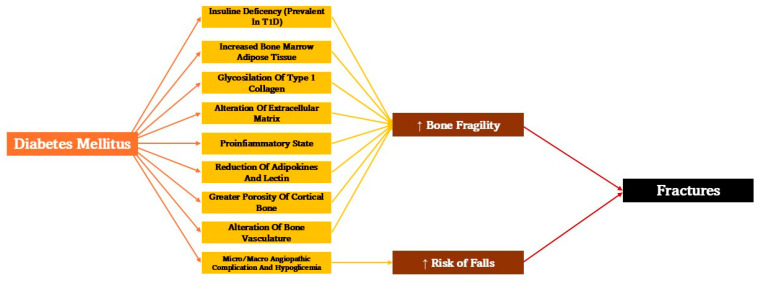
Mechanisms underlying bone loss and fractures in type 2 diabetes mellitus.

Additionally to this, the chronic micro- and macroangiopathic complications of diabetes, such as retinopathy, neuropathy, and peripheral arteriopathy, contribute to the increased fracture risk in T2D patients, as they are more prone to falling [13] (Figure 1).

### 1.2. Effect of Antidiabetic Drug on Bone Health

As regards the various anti-hyperglycemic agents, the results on their effect on bone metabolism are controversial [29].

Metformin appears to have neutral effects on bone [30] although in animal models it appears to increase bone mass by reducing the accumulation of AGEs in the bone extracellular matrix [31].

Although sulfonylureas appear to have a potential stimulating effect on osteoblast proliferation and differentiation [31], the increased fall risk associated with hypoglycaemic episodes places them, in the few studies in which they have been studied in humans, as agents that increase the risk of fracture [32].

Thiazolidinediones reduce bone formation through activation of the peroxisome proliferator-activated receptor gamma (PPARγ), resulting in promotion of osteocyte apoptosis [33].

The hypothesized presence of receptors for glucagon-like peptide 1 (GLP-1) on osteoblasts has led to several in vitro studies testing the effect receptor agonists (GLP-1RAs) may have on bone: in particular, these appear to inhibit sclerostin [34]. From a clinical point of view, however, the osteogenic effect of GLP.-1Ra has not been demonstrated and is controversial: whereas liraglutide appears to reduce fracture risk, the use of exenatide showed an increased fracture risk [35].

The effects on bone of dipeptidylpeptidase4 inhibitors (DPP4i) are also contrasting and poorly studied: while in mouse models they appeared to increase trabecular bone volume by suppressing resorption [36], a meta-analysis emphasized a neutral role of these drugs [37].

Finally, a relatively new antidiabetic drug, Tirzepatide, a dual Gastric Inhibitory Peptide GIP/GLP-1Ra, seems to have a neutral effect on bone health, although these data are limited to mouse models [37].

## 2. Methods

### Search Strategy

A scoping review of the available literature was conducted. Firstly, the studies were retrieved from the online databases PubMed, Scopus, and Web of Knowledge, by matching the following keywords: “SGLT2 inhibitors”, “diabetes mellitus”, “calcium metabolism”, “bone fragility”, and “bone turnover”. A preliminary filter on the online search was applied by language (English) and availability of full text articles. Additionally, the reference lists of the included studies were examined in order to identify further potentially relevant studies missed during the database search. The online search was definitively completed on 31 January 2025.

## 3. The Controversial Role of SGLT2i on Fracture Risk

Among the hypoglycemic drugs approved for the T2D treatment [38], SGLT2 inhibitors (SGLT2i) are gaining increasing attention due to their proven cardio-renal efficacy. Sodium-glucose cotransporter-2 inhibitors block sodium-dependent glucose transporter-2 located in the early proximal renal tubule, which is responsible for reabsorption of most (80–90%) of the glucose filtered by the glomerulus [39]. The resulting enhanced glycosuria acts by lowering plasma glucose concentrations. This mechanism of action is dependent on blood glucose levels and is independent of the action and availability of insulin. These drugs have been demonstrated to reduce HbA1c by approximately 0.6–1.0% [40].

To date, from available literature, it appears that the role of SGLT2i on bone metabolism is controversial. In fact, in some studies, their use has been reported to increase the risk of skeletal fractures [41]. This prompted the US Food and Drug Administration and Canadian Health to issue a ‘warning and caution’ about the risk of fractures, especially associated with canagliflozin [42,43].

### 3.1. Preclinical Studies

In mouse models, canagliflozin demonstrated a significant rise in markers of bone resorption, particularly carboxy-terminal telopeptide of collagen type 1 (CTX), with consequent hypercalciuria and higher circulating serum levels of Fibroblast Growth Factor 23 (FGF-23), with evident deterioration of trabecular bone mass and marked deficits in cortical and trabecular bone architecture [44,45]. Furthermore, deletion of SGLT2 in mice not only led to increased glycosuria and reduced body weight; it also showed reduced femur length and BMD in mice after 25 weeks, suggesting skeletal impairment that remained independent of circulating levels of the major markers of bone turnover, as reflected in the normal serum levels of calcium, phosphate, parathormone (PTH), vitamin D, and FGF-23 recorded in the study [46]. Similarly, using female Apoe^−/−^ mice on a high-fat diet to replicate a model of advanced vascular damage, the administration of empagliflozin 25 mg/kg body weight once daily was proved to be non-superior in improving vascular calcifications and showed a more pronounced reduction in both vertebral and femoral BMD [47].

### 3.2. Clinical Studies

**Figure 2:**
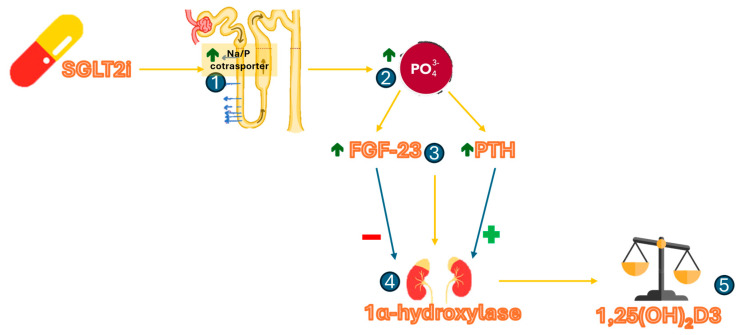
Potential effects of SGLT2i on phospho-calcium metabolism. (1) SGLT2i causes at the proximal renal tubule, through a reduction in the transport of Na+ from the lumen into the cell, an increased action of the Na/P cotransporter, which, driven by the electrochemical gradient, drags phosphorous molecules with it into the bloodstream, leading to an increase in phosphatemia (2). (3) High blood phosphorus levels then lead to increased production of FGF-23, which is secreted by osteocytes. This is presumably responsible for promoting hyperphosphaturia. Similarly, increased blood levels of phosphorus lead to a reduction in free serum ionized calcium levels; this in turn promotes activation of the parathyroid glands with subsequent overproduction of PTH. This promotes a reduction in tubular reabsorption of phosphorus. (4) At the renal level, however, the effects of FGF-23 and PTH are opposite: while PTH increases the 1-alpha-hydroxylation of inactive vitamin D, FGF-23 exerts a contrasting effect on this enzymatic activity; this induces a balance in the production of 1,25-dihydroxyvitamin D, which makes the effect of these drugs on it and thus on bone metabolism even more uncertain (5).

Published data show that SGLT2i increase serum phosphate levels [48,49], probably by acting at the renal tubular level, where the electrochemical gradient caused by sodium excretion results in increased sodium-phosphorus co-transport [50]. The increase in serum phosphorus leads by a feedback mechanism to an increased production of FGF-23 and PTH, which promote increased phosphaturia by decreasing its reabsorption. At the renal level, PTH and FGF-23 have opposing effects on the 1α-hydroxylation of 25-hydroxyvitamin D, while PTH increases its hydroxylation, FGF-23 reduces it. From existing literature, it appears that SGLT2i acts both by increasing PTH and FGF23 [48]. For this reason, it is not easy to establish what effect this class of drugs has on vitamin D hydroxylation (Figure 2). However, the available data show a small decrease in average 1,25-dihydroxyvitamin D levels [−12%] with canagliflozin [51].

Real-world evidence demonstrated that canagliflozin resulted in a reduction of total hip BMD in a cohort of T2D patients aged 55–80 years after 104 weeks of treatment, with an increase in bone resorption marker β-CTX at week 52, which was significantly correlated with weight loss; concurrently, osteocalcin, a surrogate index of bone formation, significantly increased after 52 weeks; curiously, reduced estradiol levels due to changes in body fat related to canagliflozin treatment appeared to partly contribute to the loss of BMD [52]. In contrast, Rosenstock et al. reported that ertugliflozin had no adverse effects on BMD in the lumbar spine, femoral neck, hip, and distal forearm regions after 26 weeks of treatment either in the overall population or in the cohort of postmenopausal women [53]. Similarly, in an international, multi-center, randomized, double-blind, placebo-controlled study, dapagliflozin showed no significant differences in BMD in the lumbar spine, femoral neck, hip, and marker of bone formation and resorption after 50 weeks of treatment [54].

In a population-based cohort study involving more than 3900 women over the age of 65 years divided into two groups according to the use or non-use of SGLT2i therapy, a statistically significant increase in the incidence of vertebral fractures was found in the SGLT2i users’ group, especially in older women with T2D [55].

In a Japanese quasi-experimental study involving 100 patients treated with metformin and 600 mg calcium carbonate D3 tablets. Initiation of empagliflozin 10 mg therapy led to a significant reduction in serum fasting blood glucose (FBG) levels, HbA1c, and postprandial blood glucose (2hPG), and an improvement in BMD levels and in serum calcium and phosphorus levels compared to the control group [56].

On the other hand, in a meta-analysis of 78 randomized controlled trials, for all SGLT2i, only treatment with canagliflozin was associated with a higher incidence of fractures [57]. One of the explanations that can justify this evidence could be the increased volume depletion and its consequent orthostatic hypotension with a higher risk of falls in canagliflozin users. The CANVAS study (CANagliflozin cardioVascular Assessment Study Program) [58] revealed a higher risk of low-trauma fractures and all fractures in the canagliflozin group compared to the placebo group, but the CANVAS-R study did not confirm this observation [59]. So far, there is no explanation for the differences between the two studies, considering that the population involved in the CANVAS-R program had a more impaired renal function and therefore could present a higher fracture risk [60]. So the reason for the increased risk of fractures with canagliflozin remains unknown [61]. Behind this difference that seems to distinguish canagliflozin from other SGLT2i seems to be the characteristic inhibition of SGLT1 receptors that is also peculiar for this molecule. In addition to the renal level, these receptors also appear to be present in the intestine [62], where they seem to interfere with carbohydrate absorption [63], thus reducing postprandial glycaemia and enhancing calcium absorption, resulting in hypercalcemia and absorptive hypercalciuria [64]. Thus, in rats, it was seen that such increased calcium absorption led to hyperostosis with reduction of bone turnover markers, in particular PTH and 1,25-dihydroxyvitamin D [65]. In contrast, however, two recent meta-analyses examined the impact of SGLT2i on BMD, bone metabolism markers, and fractures. None, however, showed a statistically significant worsening of any bone parameter [66,67] (Table 1).

**Figure 3:**
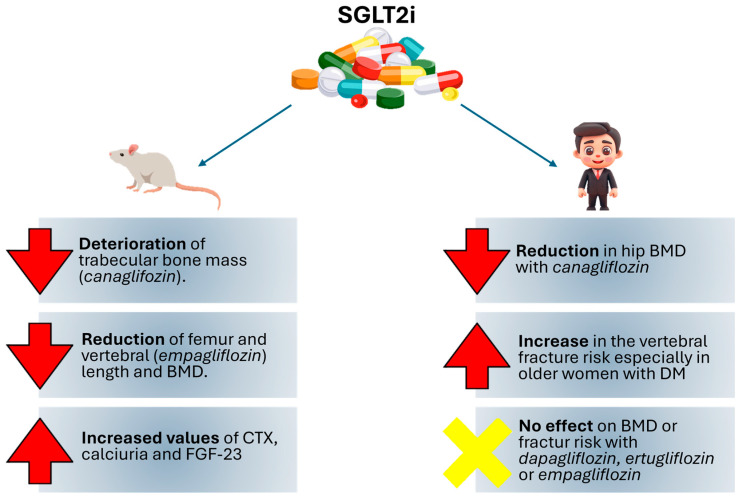
Effect of SGLT2i on both mice and human models.

A summary of the effects of SGLT2i on bone metabolism, both in mice and in human models, is reported in Figure 3.

## 4. Discussion and Limitations

SGLT2i are among the oral hypoglycemic drugs on which the scientific literature has focused the most attention due to their proven beneficial effects on various cardiological, vascular, and renal pathologies and due to their positive impact on reducing the risk of death and hospitalization from cardiovascular causes. For this reason, attention on these drugs covers several fields: one of these is related to calcium-phosphorus metabolism and bone health. However, as summarized in our review, to date results are limited and conflicting, especially about real-world studies where the effect of these molecules on bone metabolism appears to be insignificant, although some research has shown negative effects, particularly of SGLT1 inhibition. To date, however, scientific evidence is scarce and is based on studies mainly conducted in murine models, and this unavoidably represents a limitation of this review.

## 5. Conclusions

Therefore, even today the link between SGLT2i and bone metabolism remains controversial and not well elucidated, and further studies are needed to verify the long-term effects on bone health of this class of drugs.

## Figures and Tables

**Table 1:** Real-world studies on the role of SGLT2i on fracture risk.

Methods	Population	Drug	Results	Ref.
Randomized study. 26 weeks, double-blind, placebo-controlled period and a 78 weeks, double-blind, placebo-controlled extension.	716 T2D patients aged 55–80 years	Canagliflozin 100 mg or 300 mg	↓ Total hip BMD over 104 weeks ↑ Collagen type 1 β-carboxy-telopeptide at week 52 ↓ Estradiol	[38]
Double-blind, 26 weeks, multicenter study with ongoing 78 weeks extension	621 T2D patients	Ertugliflozin 5 or 15 mg	No adverse impact on BMD at week 26	[39]
International, multi-center, randomized, parallel-group, double-blind, placebo-controlled study	182 T2D patients (women 55–75 years and men 30–75 years)	Dapagliflozin 10 mg	No significant changes from baseline in P1NP, CTX, or BMD	[40]
The population-based cohort study data from the National Health Insurance Service of Korea (2013–2020)	3959 T2D women of age > 65 years	1333 patients in treatment with dapagliflozin, empagliflozin, ipragliflozin, and ertugliflozin or other drugs	↑ Risk of vertebral fracture than non-SGLT2i use in elderly women	[41]
Quasi-experimental study	100 T2D patients with osteoporosis	Empagliflozin or placebo	↑ Bone mineral density, ↑phosphorus and calcium metabolism ↓ Incidence of fracture	[42]
Multicenter, prospective, randomized, double-blind, placebo-controlled trial	10142 T2D patients	Canagliflozin or placebo	↑ Risk of low-trauma fractures and all fractures in the canagliflozin group	[44]
Multicenter, prospective, randomized, double-blind, placebo-controlled trial	5812 T2D patients	Canagliflozin 100 or 300 mg	No difference in risk factors	[45]

## Data Availability

No new data were created or analyzed in this study.

## Abbreviations

The following abbreviations are used in this manuscript:

2hPG	Postprandial blood glucose
AGEs	Advanced glycation end products
BAD	Bcl-2 agonist of cell death
BMAT	Bone Marrow Adipose Tissue
BMD	Bone mineral density
BMI	Body Mass Index
CANVAS	CANagliflozin cardioVascular Assessment Study Program
CANVAS-R	CANagliflozin cardioVascular Renal Assessment Study Program
CTX	Carboxy-terminal telopeptide of collagen type 1
FBG	Fasting blood glucose
FGF-23	Fibroblast Growth Factor 23
HbA1c	Hemoglobin A1C
IGFR-1	Insulin-like growth factor 1 receptor
PTH	Parathyrod Hormone
SGLT2i	Sodium-glucose cotransporter-2 inhibitors
T1D	Type 1 diabetes
T2D	Type 2 diabetes
